# Cross-resistance to clinically used tyrosine kinase inhibitors sunitinib, sorafenib and pazopanib

**DOI:** 10.1007/s13402-015-0218-8

**Published:** 2015-02-11

**Authors:** Kristy J. Gotink, Maria Rovithi, Richard R. de Haas, Richard J. Honeywell, Henk Dekker, Dennis Poel, Kaamar Azijli, Godefridus J. Peters, Henk J. Broxterman, Henk M. W. Verheul

**Affiliations:** Department of Medical Oncology, VU University Medical Center, Rm 3A46, De Boelelaan 1117, 1081 HV Amsterdam, The Netherlands

**Keywords:** Cross-resistance, Lysosomes, Tyrosine kinase inhibitor, mTOR inhibitor

## Abstract

**Purpose:**

When during cancer treatment resistance to a tyrosine kinase inhibitor (TKI) occurs, switching to another TKI is often considered as a reasonable option. Previously, we reported that resistance to sunitinib may be caused by increased lysosomal sequestration, leading to increased intracellular lysosomal storage and, thereby, inactivity. Here, we studied the effect of several other TKIs on the development of (cross-) resistance.

**Methods:**

TKI resistance was induced by continuous exposure of cancer cell lines to increasing TKI concentrations for 3–4 months. (Cross-) resistance was evaluated using MTT cell proliferation assays. Intracellular TKI concentrations were measured using LC-MS/MS. Western blotting was used to detect lysosome-associated membrane protein-1 and −2 (LAMP1/2) expression.

**Results:**

The previously generated sunitinib-resistant (SUN) renal cancer cells (786-O) and colorectal cancer cells (HT-29) were found to be cross-resistant to pazopanib, erlotinib and lapatinib, but not sorafenib. Exposure of 786-O and HT-29 cells to sorafenib, pazopanib or erlotinib for 3–4 months induced drug resistance to pazopanib and erlotinib, but not sorafenib. Intracellular drug accumulation was found to be increased in pazopanib- and erlotinib-, but not in sorafenib-exposed cells. Lysosomal capacity, reflected by LAMP1/2 expression, was found to be increased in resistant cells and, in addition, to be transient. No cross-resistance to the mTOR inhibitor everolimus was detected.

**Conclusions:**

Our data indicate that tumor cells can develop (cross-) resistance to TKIs, and that such resistance includes increased intracellular drug accumulation accompanied by increased lysosomal storage. Transient (cross-) resistance was found to occur for several of the TKIs tested, but not for everolimus, indicating that switching from a TKI to a mTOR inhibitor may be an attractive therapeutic option.

## Introduction

The human kinome encodes 518 protein kinases, many of which are deregulated in cancer [1]. Receptor tyrosine kinases, belonging to the vascular endothelial growth factor (VEGF) signaling pathway, serve as validated clinical anticancer targets for tyrosine kinase inhibitors (TKIs) such as sunitinib, sorafenib and pazopanib [2]. These multi-targeted TKIs have shown clinical benefit as monotherapy in renal cell cancer, and their application is currently expanding to other tumor types [3]. Despite their clinical benefit, however, complete remissions are rare and with time patients invariably suffer from disease progression, leading to discontinuation of treatment. In case of progression or unacceptable toxicity for either one of these TKIs, switching to another TKI is considered as a bona fide treatment option [4, 5]. Such sequential use can indeed be effective, i.e., pazopanib and sorafenib have shown clinical activity after sunitinib failure, suggesting that cross-resistance may be only partial [5, 6].

Currently, detailed information is lacking on patterns of resistance or cross-resistance of tumor cells to long-term exposure to multi-targeted TKIs. Contrary, single targeted TKIs, such as e.g., vemurafenib, targeting the *BRAF* ^*V600E*^ mutation in melanomas, or gefitinib, targeting activating *EGFR* mutations in lung cancer, often lead to the development of secondary drug-resistant kinase mutations thereby, at least partly, explaining the development of TKI resistance [7]. As yet, it is poorly understood how tumor cells respond to long-term kinase inhibitor exposure, e.g., to the clinical administration of sunitinib, where partial responses are most commonly observed. We have recently mimicked prolonged sunitinib exposure in vitro and showed that continuous exposure to sunitinib for several months can induce resistance to this TKI in 786-O renal cell cancer and the HT-29 colorectal cancer cell lines [8]. Importantly, when grown as in vivo xenografts in mice, the HT-29 sunitinib-resistant cell line remained resistant (unpublished result). In addition, we found that sunitinib resistance was accompanied by an increased lysosomal storage capacity and was reversible upon removal of the drug within several weeks. Therefore, this transient form of resistance may be an adaptation to (partial) inhibition of multiple kinases and/or to a partly disturbed lysosomal function, rather than a stable, genetic form of resistance. These results support other preliminary reports indicating that re-challenging of patients with sunitinib, after a certain recovery period, may be a bona fide treatment option [9, 10].

In order to obtain further insight into the possible consequences of long-term administration of sunitinib or other TKIs to the sensitivity of tumor cells to second line therapy, we explored the resistance and cross-resistance patterns of tumor cells to several multi-targeted TKIs and the mTOR inhibitor everolimus. Since there is preclinical and clinical evidence for the existence of synergistic interactions between EGFR and VEGFR inhibitors in various tumor types [11–13], we also included erlotinib and lapatinib in our current study. We found that sunitinib-resistant tumor cells are cross-resistant to some, but not all, TKIs tested in conjunction with increased intracellular drug accumulation. Upon continuous exposure of both 786-O and HT-29 cells, resistance could be induced to some TKIs, and the results are comparable to the cross-resistance findings. Furthermore, in the resistant cells the lysosomal compartment was increased as revealed by increased LAMP1/2 expression.

## Materials and methods

### Reagents

Sunitinib malate was kindly provided by Pfizer Oncology (New York, NY). Sorafenib, pazopanib, erlotinib hydrochloride, lapatinib di-*p*-toluenesulfonate and everolimus were purchased from LC Laboratories (Woburn, MA). All drugs were prepared as 20 mM stock solutions in DMSO (Sigma-Aldrich), except erlotinib hydrochloride, which was prepared as a 10 mM stock solution in 96% DMSO/4% H_2_O. All stock solutions were stored at −80 °C.

### Cell culture

786-O renal cell cancer (RCC) and HT-29 colorectal cancer (CRC) cell lines were cultured in DMEM supplemented with 5% fetal bovine serum (FBS) and maintained in a humidified incubator containing 5% CO_2_ at 37 °C. Both cell lines were purchased from the American Tissue Culture Collection (ATCC) and were authenticated by STR profiling (Baseclear, Leiden, Netherlands). The generation of the sunitinib-resistant sub-lines 786-O SUN and HT-29 SUN, continuously cultured in the presence of 5 μM or 10 μM sunitinib, respectively, has been described previously [8]. To induce resistance to sorafenib, pazopanib and erlotinib, the parental cell lines 786-O and HT-29 were continuously exposed for 3–4 months to gradually increasing concentrations of the respective drugs. The initial exposure concentrations were set between the IC_10_ and IC_50_ values for each specific compound. When cells reached confluence, they were split and the TKI concentration was at maximum doubled in the subsequent culture period. This procedure was repeated for 3–4 months, until a stable well-tolerated TKI concentration was reached and TKI sensitivity was tested as described below.

### MTT proliferation assay

The (cross-) resistance to different drugs was evaluated by MTT proliferation assays as previously described [8]. Briefly, all cells were seeded in 96-well culture plates without drug and allowed to adhere for 24 h. Subsequently, t = 0 was measured using MTT (3-(4,5-dimethylthiazolyl-2)-2,5-diophenyl tetrazolium bromide) and drugs were added at different concentrations (in 3-fold). After 96 h, proliferation was assessed using MTT. Experiments were repeated 3 times independently, unless stated otherwise.

### TKI measurements by LC-MS/MS

Parental and sunitinib, sorafenib, pazopanib or erlotinib exposed cells were seeded in 6-well culture plates and allowed to adhere for 48 h. Next, cells were washed once with PBS and incubated with TKI-containing medium as indicated. After 24 h, cells were washed three times with ice-cold PBS after which cells were trypsinized at 37 °C for 5 min and, when detached, ice-cold PBS was added. Next, samples were re-suspended, collected and counted. After centrifugation at 13,000 rpm/ 4 °C for 10 min, the supernatant was discarded and pellets were snap frozen in liquid nitrogen and stored at −80 °C until analysis.

For TKI accumulation analyses, cell pellets were reconstituted in 100 μl of Milli-Q water and homogenized by gentle aspiration. Subsequently, 20 μl of the homogeneous solution was mixed with 80 μl of ice cold acetonitrile in a round bottomed 96-well plate; a plate seal was applied to prevent evaporation. After careful ultra-sonication for 30 s, the plate was centrifuged at 4 °C/1,000 × g for 10 min and 50 μl solution was transferred to a conical 200 μl 96-well plate for injection on the optimized liquid chromatography - tandem mass spectrometry system (LC-MS/MS system). Chromatographic parameters and mass spectroscopic parameters for sunitinib, sorafenib and erlotinib were as previously reported [14]. Optimized pazopanib parameters were determined using a standard reference compound diluted to 1 μg/ml in mobile phase and linearity, accuracy and precision parameters were determined for responses across a concentration range of 5–5000 ng/ml.

### Western blot analysis

Parental and sorafenib, pazopanib or erlotinib exposed cells were seeded in TKI-containing medium and allowed to grow for 48 h. Before lysis, cells were washed twice with ice-cold PBS. Cell lysates were prepared using M-PER mammalian protein extraction reagent (Thermo Scientific) supplemented with protease and phosphatase inhibitor cocktails (Thermo Scientific). Cells were incubated with the lysis buffer mixture for 20 min on ice, scraped off, collected and, subsequently, centrifuged at 10,000 × g for 15 min at 4 °C. The supernatant was collected and stored at −80 °C until analysis. Cell lysates were prepared three times independently. Protein concentrations were determined using a micro BCA protein kit (Thermo Scientific). Samples containing 20–50 μg protein were subjected to 10 % SDS polyacrylamide gel electrophoresis and, subsequently, transferred to PVDF membranes (Immobilon-FL, Millipore). Proteins were detected using the following antibodies (catalogue numbers in parentheses): anti-LAMP-1 (sc-20011), anti-LAMP-2 (sc-18822) (Santa Cruz biotechnology) and anti-β-actin (A5441) (Sigma-Aldrich). After incubation with IRDye infrared dye labeled secondary antibodies (LI-COR Biosciences), membranes were scanned using an Odyssey Infrared Imaging System (LI-COR Biosciences). Protein expression was determined using the accompanying software program (LI-COR Biosciences) and corrected for β-actin expression. Expression levels were normalized to untreated samples.

### Statistical analyses

Data are expressed as mean ± standard error of the mean (SEM). When appropriate, results are shown as normalized data. Statistical analyses were performed using Student’s *t*-test. Resistance is defined as resistance factor > 2.5, calculated as IC_50_ value of the exposed cell line divided by the IC_50_ value of the parental cell line. A *p* value < 0.05 was considered to be statistically significant.

## Results

### TKI sensitivity of parental 786-O and HT-29 cells

**Fig. 1:**
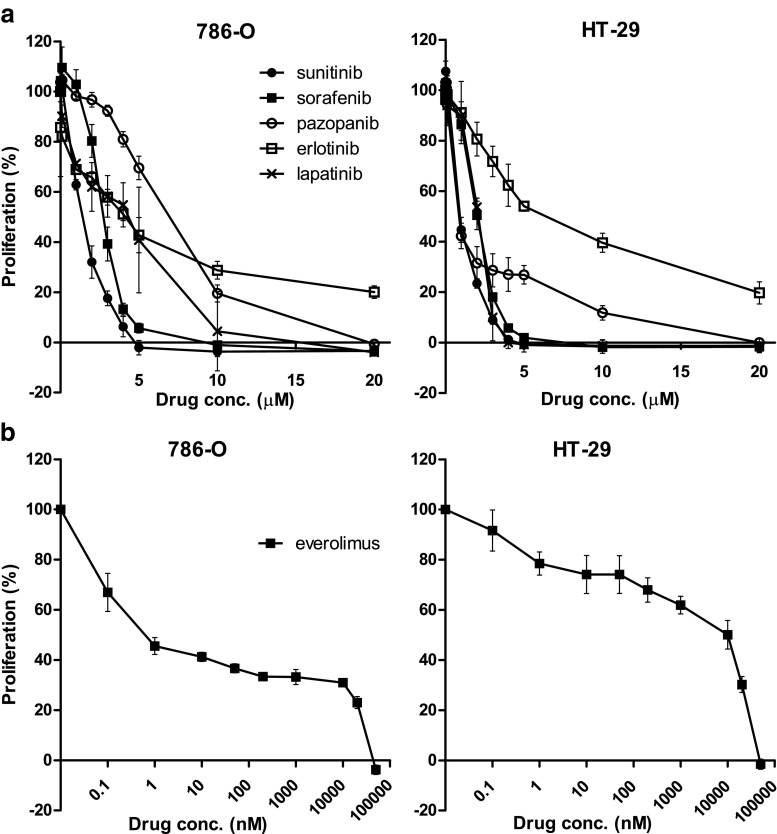
Sensitivity of (parental) 786-O and HT-29 tumor cells to different inhibitors. Proliferation assays (MTT) of 786-O (*left*) and HT-29 (*right*) parental cell lines incubated with different concentrations of (**a**) the tyrosine kinase inhibitors (TKIs) sunitinib, sorafenib, pazopanib, erlotinib or lapatinib or (**b**) the mTOR inhibitor everolimus. Results are shown as mean ± SEM

First, we determined the sensitivity of the parental cell lines 786-O PAR and HT-29 PAR to the tyrosine kinase inhibitors (TKIs) sunitinib, sorafenib and pazopanib, to the EGFR TKIs erlotinib and lapatinib and to the mTOR inhibitor everolimus in 96 h proliferation assays. The sensitivities to the respective TKIs were found to be in the same, low micro-molar range (Fig. 1a), with IC_50_ values between 0.8 and 6.5 μM (Table 1), and were comparable between the two cell lines. The mTOR inhibitor everolimus showed a different sensitivity curve compared to the TKIs and reached a plateau between ~1 nM and 10 μM, at which the proliferation hardly decreased (~IC_60_ (786-O) and ~ IC_30_ (HT29); Fig. 1b).

**Table 1:** Cross-resistance of sunitinib-resistant cells

		IC_50_ value (μM)		Resistance factor
Drug	Cell line	PAR	SUN	
Sunitinib	786-O	1.4	4.2	3.1 ***
	HT-29	0.90	3.7	4.1 ***
Sorafenib	786-O	2.7	3.4	1.2 *
	HT-29	2.0	3.7	1.8 **
Pazopanib	786-O	6.5	10	1.6
	HT-29	0.80	18	22 ***
Erlotinib	786-O	4.4	>20	>4.5 ^a^
	HT-29	6.3	>20	>3.2 ^a^
Lapatinib	786-O	4.6	6.4	1.4
	HT-29	2.1	4.2	2.0 ***
Everolimus	786-O	0.57 × 10^−3^	13 × 10^−3^	23
	HT-29	10	23	2.4 *

IC_50_ values of 786-O and HT-29 parental (PAR) and sunitinib-resistant (SUN) cell lines determined by MTT proliferation assays. IC_50_ values are shown as means (n = 2–3). Resistance factors are calculated by dividing the IC_50_ values of the sunitinib-resistant cell lines by the IC_50_ value of the parental cell line, and is denoted ‘cross-resistant’ when >2.5

*p value < 0.05; **p value < 0.01; ***p value < 0.001; ^a^, *p* value not available because IC_50_ was not reached

### TKI cross-resistance in sunitinib-resistant 786-O and HT-29 cells

**Fig. 2:**
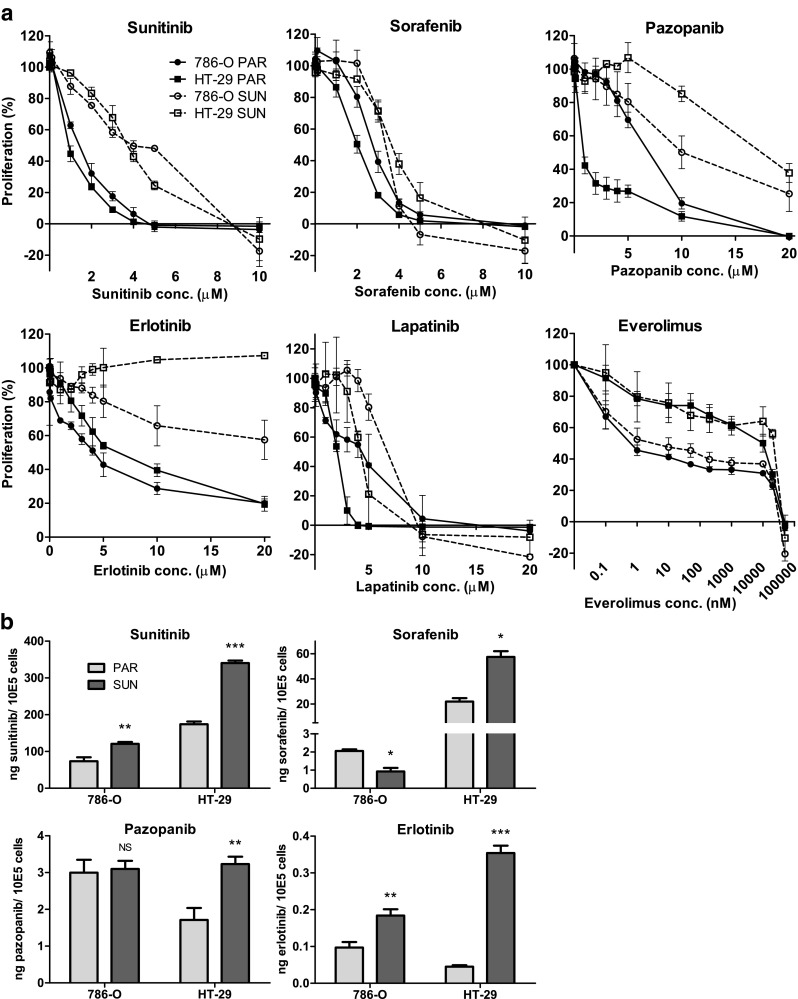
Cross-resistance of sunitinib-resistant cells. (**a**) cross-resistance patterns, determined by MTT proliferation assays, of the sunitinib-resistant 786-O SUN and HT-29 SUN cell lines to sorafenib, pazopanib, erlotinib, lapatinib or everolimus compared to parental (PAR) cell lines. (**b**) intracellular accumulation of sorafenib, pazopanib or erlotinib in parental (PAR) and sunitinib-resistant (SUN) cells. Cells were incubated for 24 h with drug-containing medium at the ~ IC_50_ concentration of the parental cell line (except sunitinib itself). Drug concentrations: sunitinib: 5 μM (786-O) or 10 μM (HT-29); sorafenib: 2 μM (both cell lines); pazopanib: 4 μM (786-O) or 2 μM (HT-29); erlotinib: 5 μM (*both cell lines*). Results are shown as mean ± SEM (*n* = 2–3). *, *p* value < 0.05; **, *p* value < 0.01; ***, *p* value < 0.001

First, we confirmed our previously reported [7] sunitinib-resistance in the 786-O SUN and HT-29 SUN cell lines with a resistance factor (RF) of 3–4 fold (Fig. 2a and Table 1). Subsequently, we determined the sensitivity of 786-O SUN and HT-29 SUN cells to sorafenib, pazopanib, erlotinib, lapatinib and everolimus (Fig. 2a and Table 1). Erlotinib showed a pronounced cross-resistance in both 786-O SUN and HT-29 SUN cells (RF ≥ 4.5 and > 3.2, respectively) and did not reach an IC_50_ within the tested concentration range. Pazopanib showed cross-resistance in HT-29 SUN cells (RF = 22), but not in 786-O SUN cells (RF = 1.6). Two other TKIs, sorafenib and lapatinib, showed no cross-resistance in the sunitinib-resistant cells. The effect of everolimus on cell proliferation was modest over a wide concentration range in the parental cells, with no detectable cross-resistance in the sunitinib-resistant cell lines.

Since we previously showed [8] that sunitinib-resistance may be associated with an increased intracellular accumulation of the drug, we also measured intracellular sorafenib, pazopanib and erlotinib concentrations in the SUN compared to the PAR cell lines (Fig. 2b). First, we confirmed that the total intracellular accumulation of sunitinib was increased in the resistant cells. Next, we found that the accumulation of erlotinib was also increased (2–8 fold higher in the sunitinib-resistant cell lines). Pazopanib showed a 2-fold higher accumulation in HT-29 SUN cells compared to HT-29 PAR cells, but a similar accumulation in 786-O SUN and PAR cells. Compared to their respective parental cells, sorafenib showed a 3-fold higher accumulation in HT-29 SUN cells, but a lower accumulation in 786-O SUN cells.

### Induction of multiple TKI cross-resistances in 786-O and HT-29 cells

**Fig. 3:**
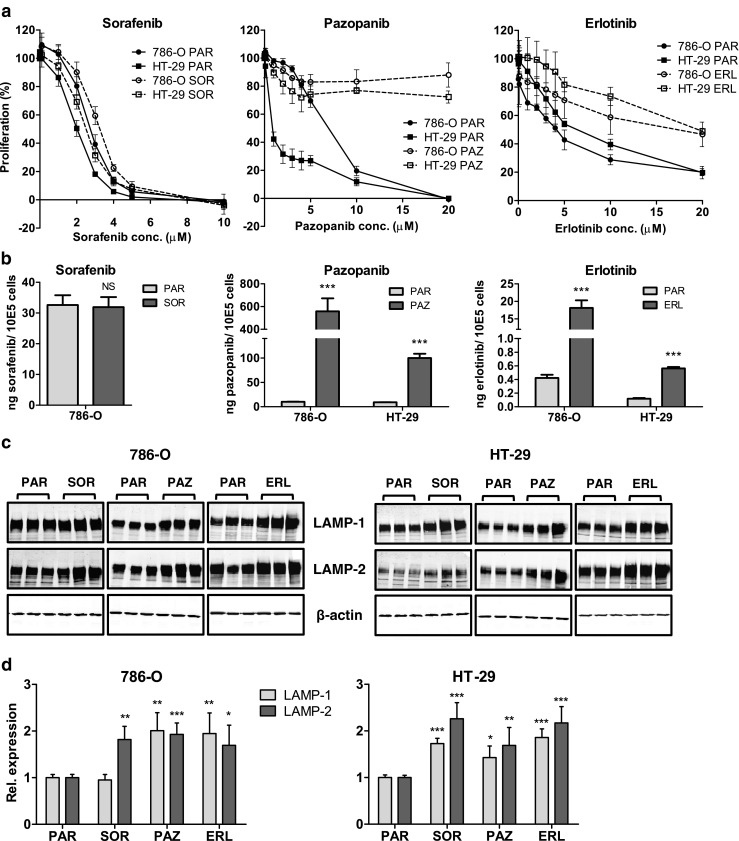
Induction of resistance to sorafenib, pazopanib and erlotinib. (**a**) resistance patterns, determined by MTT proliferation assays, of the 786-O and HT-29 cells continuously exposed to sorafenib (SOR), pazopanib (PAZ) or erlotinib (ERL) for 3–4 months. (**b**) intracellular accumulation of sorafenib, pazopanib or erlotinib in parental (PAR) and continuously exposed SOR, PAZ and ERL cells. PAR cells were incubated for 24 h with drug-containing medium at the concentration of the continuous exposure. Drug concentrations: sorafenib: 3 μM; pazopanib: 20 μM; erlotinib: 20 μM. (**c**) Western blot analysis of lysosome-associated membrane protein-1 and −2 (LAMP-1 and −2), as a measure of the lysosomal compartment. (**d**) quantification of LAMP-1 and −2 by Western blot analysis. LAMP-1 and LAMP-2 expression was corrected for β-actin expression, and normalized to untreated samples (PAR). *P* values are derived from comparison to the PAR cell line. Results are shown as mean ± SEM. *, *p* value < 0.05; **, *p* value < 0.01; ***, *p* value < 0.001

In order to investigate whether the resistant phenotype induced by sunitinib may represent an universal resistance mechanism, we exposed 786-O PAR and HT-29 PAR cells continuously for 3–4 months to increasing concentrations of the multi-targeted TKIs sorafenib and pazopanib and to the single-targeted TKI erlotinib to compare resistance induction to an EGFR family TKI. The induction of resistance by these three TKIs was found to be comparable to their cross-resistance phenotype in the sunitinib-selected cells: a prominent resistance to pazopanib and erlotinib (RF ≥ 3.1 - > 25) and no development of resistance to sorafenib (RF = 1.2; Fig. 3a and Table 2). The final concentration after 3–4 months of exposure was 3 μM sorafenib and 20 μM pazopanib or erlotinib, respectively. Higher concentrations of pazopanib and erlotinib could not be achieved due to their limited solubility in culture medium. Pazopanib- or erlotinib-resistant cells, as well as sorafenib-exposed cells, did not show cross-resistance to any of the other drugs tested (i.e., sunitinib, sorafenib, pazopanib, erlotinib, lapatinib, everolimus; data not shown). The intracellular accumulation of the selected TKIs (sorafenib, pazopanib, erlotinib) was measured in these continuously exposed cells and compared to shortly exposed parental cells. By doing so, a ~ 5–50 fold increase in pazopanib and erlotinib content, but not in sorafenib content, was found in the continuously exposed cells compared to the parental cells (Fig. 3b).

**Table 2:** Induction of resistance to sorafenib, pazopanib or erlotinib

		IC_50_ value (μM)		Resistance factor
Drug	Cell line	PAR	SOR/PAZ/ERL	
Sorafenib	786-O	2.7	3.2	1.2 *
	HT-29	2.0	2.5	1.2 *
Pazopanib	786-O	6.5	>20	>3.1 ^a^
	HT-29	0.80	>20	>25 ^a^
Erlotinib	786-O	4.4	>20	>4.5 ^a^
	HT-29	6.3	>20	>3.2 ^a^

IC_50_ values of 786-O and HT-29 parental (PAR) and sorafenib (SOR), pazopanib (PAZ) or erlotinib (ERL) selected cell lines determined by MTT proliferation assays. IC_50_ values are shown as means. Resistance factors are calculated by dividing the IC_50_ value of the inhibitor selected cell line by the IC_50_ value of the parental cell line, and is denoted ‘resistant’ when >2.5

*, *p* value < 0.05; ^a^, *p* value is not available because IC_50_ was not reached

As a measure for the lysosomal compartment the lysosome-associated membrane proteins LAMP-1 and LAMP-2, which are the most abundant constituents of the lysosomal membrane [15], were measured by Western blotting. These proteins were previously found to be increased in the sunitinib-resistant cells and to be reverted to control levels when cells became sensitive again [8]. Through Western blot analysis, we found that both LAMP-1 and LAMP-2 were expressed at increased levels in most of the continuously exposed cell lines tested (Fig. 3c). Quantification revealed a 1.5 - 2 fold increase in LAMP-1 and LAMP-2 expression levels for the three TKIs in the continuously exposed 786-O and HT-29 cell lines, except for the 786-O SOR cell line in which LAMP-1 expression was found to be unaltered (Fig. 3d).

### Pazopanib resistance induction and reversal in 786-O and HT-29 cells

**Fig. 4:**
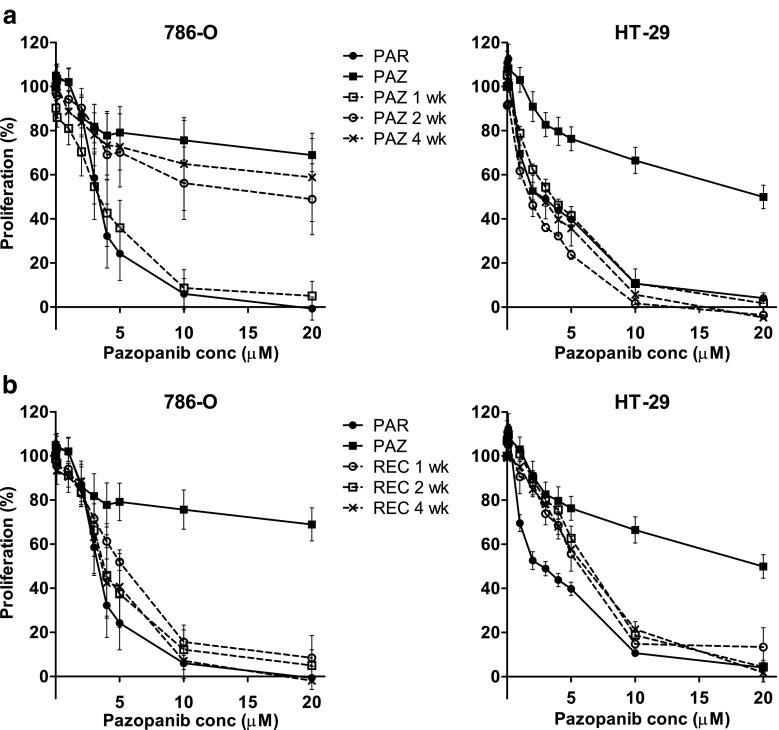
Induction and recovery of pazopanib-resistance. (**a**) induction: sensitivity to pazopanib after 1, 2 or 4 weeks of culturing the parental cells (PAR) in the presence of the drug, compared to the pazopanib-resistant cell line (PAZ; cultured for 3–4 months). (**b**) recovery: sensitivity to pazopanib 1, 2 or 4 weeks after drug withdrawal from the pazopanib-resistant cells (PAZ; cultured for 3–4 months), compared to the parental cells (PAR). Results are shown as mean ± SEM

The remarkable pazopanib-resistance, which was observed after 3–4 months continuous exposure to this drug, is of particular interest because of its increasing clinical use [2]. Therefore, additional experiments aimed at establishing a time-course for resistance induction and its subsequent reversibility were performed. To this end, 786-O and HT-29 cells were exposed to pazopanib for 1, 2 and 4 weeks. We found that resistance to pazopanib developed rapidly in 786-O cells and was fully present after a 2 week exposure, whereas such resistance developed slower in HT-29 cells (Fig. 4a). When pazopanib was removed from the resistant cells (PAZ; cultured for 4 months in presence of the drug), sensitivity was determined after 1, 2 and 4 weeks of withdrawal. The recovery was found to be rapid in both cell lines and to be nearly complete within 1 week (Fig. 4b).

## Discussion

Previously, we reported in vitro induction of resistance to sunitinib after prolonged, continuous exposure of 786-O and HT-29 cancer cell lines to this tyrosine kinase inhibitor (TKI) [8]. Induction of resistance was accompanied by an increased intracellular accumulation of sunitinib and an increase of the lysosomal compartment, as indicated by an increased expression of the lysosome-associated membrane proteins LAMP-1 and LAMP-2. The resistance phenotype was found to be transient and reversible upon removal of sunitinib (8–12 weeks). In the present study, we aimed to determine whether sunitinib-resistant cancer cell lines may exhibit cross-resistance to other targeted agents, and whether resistance induction in this experimental context is a common feature of TKIs. We tested pazopanib, sorafenib and everolimus, as TKIs that are used interchangeably or consecutively for the treatment of renal cell cancer and two other TKIs with distinct modes of action, i.e., lapatinib and erlotinib. As in our previous reports, we used the 786-O renal cell cancer (RCC) cell line, representative of a tumor type in which sunitinib, sorafenib and pazopanib exhibit single agent activities, and the HT-29 colorectal cancer (CRC) cell line which represents a tumor type in which these three drugs exhibit activity at best in a small subset of patients. This latter observation underscores the need to understand in depth the effect of prolonged administration of these agents, also in CRC [16]. We found cross-resistance of the sunitinib-resistant cell lines to the TKIs pazopanib and erlotinib, but not to sorafenib, lapatinib or everolimus. A similar resistance pattern was observed when the parental cell lines were exposed for several months to sorafenib, pazopanib or erlotinib, i.e., induction of resistance to pazopanib or erlotinib was found, but not to sorafenib. The development of (cross-) resistance was accompanied by an increased intracellular accumulation of the respective drugs and an elevated expression of the lysosomal membrane proteins LAMP-1 and LAMP-2 in the resistant cells, suggesting an involvement of the lysosomal compartment in the respective cellular adaptations. In addition, we found that resistance to pazopanib could be rapidly induced (in 786-O cells) and reversed within a few weeks after drug withdrawal.

Considering the potential mechanism(s) causing resistance to TKIs in cancer, we selected tumor cell lines and clinically approved agents to mimic the long-term use of these agents and the resulting resistance patterns observed in patients. In a clinical setting, these drugs are often applied continuously, over prolonged periods of time, of which RCC constitutes a prototype example. It is commonly assumed that mutations arising under selective pressure during therapy account for the development of acquired resistance. However, in contrast to e.g., chronic myeloid leukemia (CML) and gastrointestinal stromal tumors (GIST), where oncogene addiction is clearly at work, no resistance mutations have been discovered in primary RCCs or in RCC-derived cell lines that would constitute indisputable proof that this mutated kinase is required for tumor growth and/or survival [17]. Although sunitinib is used clinically to treat c-KIT driven GISTs, efficient growth inhibition of HT-29 CRC cells or xenografts requires a simultaneous block of EGFR [18, 19] or c-MET [20] kinases, in addition to BRAF^V600E^, consistent with the very low response rate (about 5 %) in BRAF-mutant CRCs. Although the delineation of resistance mechanisms to VEGF inhibitors is an intense field of investigation, it appears plausible that adaptation to treatment, including up-regulation of distinct angiogenic mediators [21] and reversible epithelial to mesenchymal transition (EMT) [22], may be relevant to this group of agents. Our results do support this notion, since resistance was found to be temporary and to be preserved only under continued drug exposure. Upon removal, the cells rapidly recovered their original sensitivity.

At the growth inhibitory IC_50_ of sunitinib (1–2 μM) several major downstream substrates of (receptor) tyrosine kinases are known to be inhibited, such as p-Akt(Ser473) and/or p-ERK1/2(Thr202/Tyr204) and/or p-STAT3(Tyr705) [23]. Given the apparent lack of one single targetable kinase sufficient to cause effective growth inhibition in the cell lines tested, it is plausible that a more general low level inhibition of several upstream receptors and/or non-receptor kinases converges to the downstream effects of multi-targeted TKIs like sunitinib and sorafenib [24]. A number of kinases, which have in comprehensive in vitro kinase catalytic activity assays been found to be moderately inhibited by sunitinib [25], are highly phosphorylated in HT-29 and 786-O cells, including Axl and RSK4 [20, 26]. Therefore, combined inhibition of these, as well as a number of other abundant kinases such as AMPK [27] or possibly high affinity kinases expressed at a low level such as CSFR1 [28], could lead to disruption of proper downstream survival signals. Similar to sunitinib, these considerations also apply to sorafenib and pazopanib, which have a partially overlapping kinase inhibition profile [25, 29]. Knowledge of adaptations in these or other kinase activity profiles, and on the possibility of kinome reprogramming contributing to loss of sensitivity to these TKIs, requires further phospho-proteomic based studies. Such studies may lead to insight in promising combination therapies to improve response rates, for instance by direct modulation of the Akt/mTOR signaling pathway [30].

We found that resistance induction in 786-O and HT-29 parental cells to pazopanib and erlotinib bears characteristics resembling resistance induction to sunitinib, i.e., increased intracellular drug accumulation and increased levels of LAMP-1 and LAMP-2 in the resistant cell lines. Pazopanib and erlotinib belong to the same class of hydrophobic, membrane-permeable weak bases as sunitinib, properties that could facilitate the accumulation of their protonated form in the acidic lysosomes, thereby resulting in increased accumulation and an increase in the lysosomal compartment. A recent study confirmed the lysosomal accumulation of sunitinib and revealed a disturbed intra-lysosomal pH, leading to leakage of lysosomal proteases into the cytosol [31]. As such, this mechanism may be implicated in cell death induction by this type of TKIs. An increased stabilization of lysosomes by increased LAMP-1 and LAMP-2 expression may contribute to the resistance observed. Taking a closer look, however, some differences between sunitinib-resistance and pazopanib- or erlotinib-resistance do appear. First, pazopanib- or erlotinib-resistant cells do not show cross-resistance to any of the other drugs tested (data not shown), while sunitinib-resistant cells are cross-resistant to pazopanib and erlotinib. In addition, pazopanib-resistant cells regain sensitivity much faster (~one week after drug removal) than sunitinib-resistant cells. Differences in time-to-induction of resistance may affect the outcome (4 months for pazopanib- or erlotinib-resistance versus more than one year for sunitinib-resistance). Drug characteristics and metabolism may, however, offer alternative explanations, i.e., resistance may also be influenced by the efficiency of drug efflux by drug transporters, such as P-glycoprotein, and the mutation-inducing properties of drugs [32]. For instance, though sunitinib and doxorubicin exhibit very similar physicochemical properties facilitating lysosomal accumulation, and doxorubicin intercalates into the DNA causing mutations, sunitinib as TKI affects survival signaling pathways, leading to both similar, but also distinct, cellular adaptations and resistance mechanisms [33]. Our observation that resistance to sorafenib did not develop in these cell lines under similar conditions indicates that subtle changes in physicochemical properties of a particular drug, in combination with a different kinase inhibition profile, may result in differential resistance patterns. We found that the lysosomal compartment, as measured by LAMP1/2 expression, was not or only moderately induced by sorafenib in the present schedule, and that no intracellular accumulation of sorafenib was detected after continuous exposure. Both findings support an alternative resistance mechanism for sorafenib compared to the other TKIs. A recent study showed that sorafenib resistance in hepatocellular carcinoma cells may at least partly be related to p38α (MAPK14)-dependent MEK-ERK activation, but other downstream proteins, such as STAT3, also seem to be important [34, 35]. Since sensitivities to both sorafenib and everolimus are retained in sunitinib-resistant and pazopanib-resistant cells, switching to these drugs is a reasonable strategy when progression occurs in patients treated with sunitinib or pazopanib. The observation that sunitinib-resistant cells do not show cross-resistance to sorafenib is consistent with results from clinical trials that revealed a survival benefit for sorafenib in a second-line setting in patients who progressed upon sunitinib treatment [36]. The mTOR inhibitor everolimus may, based on the differences between these two classes of drugs (multi-targeted TKI versus mTOR inhibitor), be an even more interesting candidate for sequential therapy. In the past, everolimus has been shown to prolong progression-free survival after failure of VEGF-targeted therapy [37]. Furthermore, everolimus is currently assessed as alternate treatment with pazopanib in a rotating schedule in patients with RCC [38].

In conclusion, we found that tumor cells can develop (cross-) resistance to TKIs, such as sunitinib, pazopanib and erlotinib, which is accompanied by an increased intracellular accumulation. In resistant cells, an increased lysosomal compartment was found that may cause lysosomal drug sequestration and, thereby, an increased intracellular accumulation that may prevent intracellular drug activity. The notion that prolonged administration of these TKIs may cause tumor cell adaptations and (cross-) resistance to some, but not all, agents tested is of relevance, since these agents are frequently considered for combination or sequential therapy. In addition, lysosomal protein expression could serve as a candidate biomarker for these forms of drug resistance.
